# Mouse and Fly Sperm Motility Changes Differently under Modelling Microgravity

**DOI:** 10.3390/cimb43020043

**Published:** 2021-07-05

**Authors:** Irina V. Ogneva

**Affiliations:** 1 Cell Biophysics Laboratory, State Scientific Center of the Russian Federation Institute of Biomedical Problems of the Russian Academy of Sciences, 76a, Khoroshevskoyoe shosse, 123007 Moscow, Russia; iogneva@yandex.ru; Tel.: +74-99-1956398; Fax: +74-99-1952253; 2 Medical and Biological Physics Department, I. M. Sechenov First Moscow State Medical University, 8-2 Trubetskaya St., 119991 Moscow, Russia

**Keywords:** weightlessness, evolution, sperm motility, regulation of motility, phosphorylation

## Abstract

Sperm motility is essential for the natural fertilization process in most animal species. Despite the fact that evolution took place under conditions of constant gravity, the motility of spermatozoa of insects and mammals under microgravity conditions changes in different ways. In this work, an attempt was made to explain this effect. The sperm motility of the fruit fly Drosophila melanogaster and the mouse was evaluated after exposure to a random positioning machine for 6 h. Sodium fluoride was used to inhibit serine/threonine phosphatases, sodium orthovanadate was used to inhibit tyrosine phosphatases, and 6-(dimethylamino)purine was used to inhibit protein kinases. The results obtained indicate that simulated microgravity leads to an increase in the speed of movement of fly spermatozoa by 30% (*p* < 0.05), and this effect is blocked by sodium fluoride. In contrast, a 29% (*p* < 0.05) decrease in the speed of movement of mouse spermatozoa under simulated microgravity is prevented by 6-(dimethylamino)purine. Moreover, after 6 h of exposure, the content of tubulin cytoskeleton and actin proteins remains at the control level in the spermatozoa of flies and mice. However, the content of the actin-binding protein alpha-actinin in fly sperm decreases by 29% (*p* < 0.05), while in mouse sperm, the relative content of alpha-actinin1 increases by 94% (*p* < 0.05) and alpha-actinin4 by 121% (*p* < 0.05) relative to the control, as determined by 6 simulated microgravity tests. It can be assumed that the effect of simulated microgravity on the motility of mammalian spermatozoa is mediated through the regulation of phosphorylation and that of insects through the regulation of dephosphorylation of motor proteins; moreover, the development of a response to changes in external mechanical conditions has a different time scale.

## 1. Introduction

The motor activity of spermatozoa, which is a prerequisite for successful fertilization in most animal species, is provided by the structure of the cytoskeleton of their tails, called the axoneme. The axoneme is a system of microtubules formed by tubulin heterodimers according to the 9 + 2 scheme, of which 2 are central singlet microtubules, and 9 are peripheral doublets (A and B microtubules). The plus ends of the microtubules are directed towards the tip of the flagellum, and the minus ends are directed towards its base. Adjacent sets of doublets are linked by two sets of dynein molecules, which are called internal and external handles [1]. Dynein is a motor protein that moves towards the minus end of microtubules due to hydrolysis of ATP, ensuring the movement of the flagellum [2]. It should be noted that almost all proteins that form the axoneme are highly conserved in evolution, and the flagella axoneme itself is similar in the order from Chlamydomonas to humans [3]. In the region of the central microtubules, there are several kinases and phosphatases, in particular, casein kinase 1(CK1), protein kinase A (PKA) and protein phosphatase (PP) PP1, PP2A, which, by activation and inactivation of dynein, regulate the parameters of motor activity [4,5]. However, despite the high homology of structural proteins and the constant gravity during the evolutionary process, with changes in gravity, the motor activity of spermatozoa in lower and higher animals changes in different ways.

In the sea urchin, under microgravity conditions, the motor activity of spermatozoa significantly increases, which is probably associated with an increase in the level of phosphorylation of phosphotreonine- and phosphoserine-containing proteins [6,7], and the role of phosphatases is more likely due to the lack of effect with the addition of a protein kinase A inhibitor [7]. Our data obtained in the study of the motility of spermatozoa of the fruit fly Drosophila melanogaster indicate that the speed of movement of the tip of the spermatozoon’s tail when exposed under simulated microgravity after 6 h increases by 34% compared to the control group [8].

However, in mammalian experiments, the trends were different. With antiorthostatic suspension of mice for 7 days, it was shown that the proportion of motile spermatozoa did not change, but the average speed of their movement was significantly reduced [9]. After 30 days of suspension against the background of a tendency to decrease in speed, the proportion of motile spermatozoa significantly decreased [10]. In experiments with isolated spermatozoa from mice, it was also shown that 6-h exposure on a random positioning machine led to a decrease in the proportion of motile spermatozoa by almost two times compared to static and dynamic controls for the same time [11]. Human sperm motility also decreased both in experiments using a clinostat and in parabolic flights compared with the corresponding controls [12].

Comparing the results obtained in higher and lower animals, it can be assumed that the regulation of motor activity under microgravity conditions may be different. We hypothesized that despite the high conservation of the proteins that form the axoneme, protein kinases and phosphatases that regulate the phosphorylation of axonemal proteins under microgravity conditions may be different from an evolutionary perspective. Therefore, using the databases CilaDataBase (http://cildb.i2bc.paris-saclay.fr, accessed on September–November 2020), HomoloGene NCBI (https://www.ncbi.nlm.nih.gov/homologene, accessed on September–November 2020), Taxonomy Search NCBI (https://www.ncbi.nlm.nih.gov/Taxonomy/Browser/wwwtax.cgi, accessed on September–November 2020), and FlyBase (http://flybase.org, accessed on September–November 2020), we analysed homology between phosphorylation regulators in spermatozoa of flies and mice and summarize these results in the discussion. Using inhibitors (sodium orthovanadate (Na_3_VO_4_) of tyrosine phosphatases, sodium fluoride (NaF) for serine/threonine phosphatases, and 6-(dimethylamino)purine (6-DMAP) for a wide range of kinases), we showed that under microgravity, changes in the motor activity of higher and lower animals can be associated with differential regulation of the dynamic equilibrium of phosphorylation-dephosphorylation.

## 2. Materials and Methods

### 2.1. Experimental Design

Microgravity conditions were created using a random positioning machine (RPM), which provides 3D multidirectional rotation relative to the gravity vector, so that the superposition of the orientation vectors of objects in the gravity field on average per minute is zero [13].

In the experiment, we used 2-day-old virgin male adults of the fruit fly Drosophila melanogaster of the Canton S line, from which the testes were isolated and dissected in physiological saline as described earlier [8]. Analogously, for mouse sperm collection, both caudal epididymises from 21 C57balck6 male mice 12 weeks old (after euthanizing the animals by overdose isoflurane) were used and received sperm as described earlier [10].

Then, the spermatozoa were randomly assigned to study groups: C—control group, sµg—simulated microgravity, the group which was exposed on a random positioning machine for 6 h. An inhibitory assay was used to analyse the role of phosphorylation in sperm motility. In half of the samples, the inhibitor was added before the start of exposure and half after the end of exposure for 10 min. Sodium orthovanadate Na_3_VO_4_ (CAS 13721-39-6, Calbiochem, San Diego, CA, USA) at a concentration of 200 μM was used as an inhibitor of tyrosine phosphatases, sodium fluoride was used as an inhibitor of serine/threonine phosphatases (# 01148, Sigma-Aldrich, Merck, St Luis, MO, USA) at a concentration of 5 mM, and 6- (dimethylamino) purine (6-DMAP) (ab145307, Abcam, Cambridge, UK) at a concentration of 0.5 mM as a broad-spectrum kinase inhibitor.

Then, sperm motility was assessed in all samples. The remaining parts of the samples, which were incubated with the inhibitor for 6 h, as well as the corresponding control samples were frozen for subsequent protein isolation to assess the content of a number of cytoskeletal proteins.

All the experimental procedures were approved by the Commission on Biomedical Ethics of the Institute of Biomedical Problems (IBMP), the State Scientific Center of the Russian Federation and the Federal State Budgetary Institution of Science (Minutes No. 521 dated 25 September 2019). The study was carried out in compliance with the ARRIVE guidelines.

### 2.2. Estimation of the Sperm Motility

The procedure for estimating sperm motility in Drosophila melanogaster and mice was described in detail earlier [8,10]. Briefly, a drop of the sperm suspension was applied to a Makler chamber (Sefi Medical Instruments Ltd., Haifa, Israel) and observed under a phase-contrast microscope (Eclipse E200 MV, Nikon, Tokyo, Japan) at a magnification of 200×. For sperm motility analysis, we made video recordings with a Basler puA1600-60uc colour camera with an e2V EV76C570 CMOS sensor and 60 frames per second with 2 megapixel resolution (Basler AG, Ahrensburg, Germany). Motility data were analyzed in the ImageJ program using the plugin Tracking in Fiji software (version for Windows, https://imagej.net/Fiji, accessed on September–November 2020). To calculate the speed (µm/s) for flies, we used the end of the tail of the sperm; for mice, the head of the sperm was monitored, and the distance it travelled per second was measured.

### 2.3. Western Blotting

Frozen cells were homogenized in Laemmli buffer containing a protease inhibitor cocktail (#1862209, Thermo Scientific™, Waltham, MA, USA). Protein concentration was measured and based on these data an equal amount of protein was placed in each well, and separated by denaturing electrophoresis on polyacrylamide gels (Bio-Rad Laboratories, Hercules, CA, USA), and transferred to a nitrocellulose membrane [14]. The specific primary antibodies were used at the dilutions recommended by the manufacturers to determine the levels of proteins. For alpha-tubulin (Tuba1c, 50 kDa), beta-tubulin (Tubb4b, 50 kDa) and beta-actin (Actb, 42 kDa) we used rabbit antibodies (respectively, #ab52866, #ab179513 and #ab227387, Abcam, Cambridge, UK). For dynein (axonemal intermediate chain, Dnaic1, 74 kDa) were used mouse antibodies (#14-9772-80, Invitrogen by Thermo Fisher Scientific, Waltham, MA, USA, eBioscience^TM^). For detection the mouse gamma-actin (Actg, 42 kDa), alpha-actinin1 (Actn1, 103 kDa) and alpha-actinin4 (actn4, 102 kDa) we used mouse antibodies (respectively, #sc-65638, #sc-17829 and #sc-393495, Santa Cruz Biotechnology, Inc., Dallas, TX, USA). For Drosophila alpha-actinin (Actn, 103 kDa) we used primary antibodies, provided in rat (#ab50599, Abcam, Cambridge, UK).

HRP-linked horse and goat antibodies for chemiluminescent detection were used as secondary antibodies to detect mouse IgG and rabbit IgG accordingly (#7076S and #7074S Cell Signalling Technology, Inc., Danvers, MA, USA) at a dilution of 1:10,000. These membranes were then treated with SuperSignal™ West Femto Maximum Sensitivity Substrate (#34096, Thermo Scientific™, Waltham, MA, USA) at a dilution of 1:10. The protein bands were revealed using the Gel Doc XR+ System (Bio-Rad Laboratories, Hercules, CA, USA).

Biotinylated goat antibodies (#B7139, Sigma, Germany) were used as the secondary antibodies to detect primary rat antibodies against Drosophila Actn at a dilution of 1:10,000, and the membranes were then treated with streptavidin solution conjugated with horseradish peroxidase (Sigma, Germany, #E2886) at a dilution of 1:10,000. The protein bands were revealed using 3,3’-diaminobenzidine (#E733, Amresco, VWR Life Science, Solon, OH, USA).

The western blotting data were analysed in the ImageJ program.

### 2.4. Statistical Analysis

The results were statistically analyzed by ANOVA using the post hoc t-test with a significance level of *p* < 0.05 to assess the reliability of differences between groups. The data are presented as the mean ± standard error of the mean (M ± SE).

All the methods were carried out in accordance with the relevant guidelines and regulations.

## 3. Results

### 3.1. Database Search

According to the UniProt database (https://www.UniProt.org, accessed on September–November 2020), dynein has a large number of phosphorylation sites, both for tyrosine and for serine and threonine, which significantly increases the number of possible regulatory pathways. Analysis of the results of proteomic studies presented in CilaDataBase (http://cildb.i2bc.paris-saclay.fr, accessed on September–November 2020) indicates a few screening studies of the proteome of fruit fly spermatozoa [15,16] and mice [17]. However, for mouse spermatozoa, the tail proteome is presented separately [18]. We focused on the phosphorylation regulators that were found in these screening studies, as well as those that were mentioned in the literature. Then, using FlyBase (http://flybase.org, accessed on September–November 2020) or HomoloGene NCBI (https://www.ncbi.nlm.nih.gov/homologene, accessed on September–November 2020), we looked for the corresponding homologue or orthologue in the fly, if it is mentioned in the literature in the mouse, and vice versa.

According to the database search results, serine and threonine phosphorylation regulators have the highest representation in spermatozoa. Protein kinase A is present in both the fly and mouse, but its beta isoform, PKAβ, identified in mouse sperm [17], does not have an orthologue in the fly, similar to A kinase (PRKA) anchor proteins 3 and 4, which have been found in the tails of mouse sperm [18]. Similarly, glycogen synthase kinase 3 alpha GSKα [19,20], misshapen-like kinase 1 (zebrafish) MINK1, and WNK lysine deficient protein kinase 2 WNK2 were found in the sperm of mice, along with calcium/calmodulin-dependent protein kinase IV CAMK4 [17]; however, we did not find their orthologues in flies when searching in the FlyBase and HomoloGene databases. At the same time, Ser/Thr phosphatases described in mouse spermatozoa practically all have homologues in the fly. Moreover, for PP1γ phosphatase, which is most widely represented in the sperm of mice [17,21,22], there is not one but three homologues in flies, two of which were detected in spermatozoa [15]. However, the dual specificity phosphatases DUSP3 and DUSP4, which are found in the spermatozoa of the sea urchin [23] and some mammals [24], have not been described in the spermatozoa of mice, although expression in the tissues of the testes takes place (Gene NCBI https://www.ncbi.nlm.nih.gov/gene/, accessed on September–November 2020), and flies do not have orthologous genes.

The regulators of tyrosine phosphorylation in spermatozoa are not so diverse and, in general, rather conservative. The kinases of the SRC family described in spermatozoa of mice [25,26,27] and fer (fps/fes related) tyrosine kinase FER [28] have orthologues in the fly (HomoloGene). We were unable to find in the fly the orthologue of CABYR—calcium binding tyrosine- (Y) -phosphorylation regulated kinase, identified in mouse spermatozoa [17]. In addition, one of the non-receptor phosphatases, PTPN1 (PTP1B), in mouse spermatozoa [24,29] also lacks an orthologue in *Drosophila melanogaster*.

Notable is the presence of three proteins in the fly, which are orthologous to one isoform of phosphohistidine phosphatase 1 PHPT1 of the mouse. According to the HomoloGene NCBI data, only one of these proteins, janA (NP_788763.1), is a homologue of mouse Phpt1 (NP_083569.1), which is expressed in both males and females. Two other proteins, ocn (NP_524578.1) and janB (NP_476584.2), have been identified in the spermatozoa of *Drosophila melanogaster* [15], and it is known that janB is expressed only in males FlyBase [30].

### 3.2. Sperm Motility

**Figure 1:**
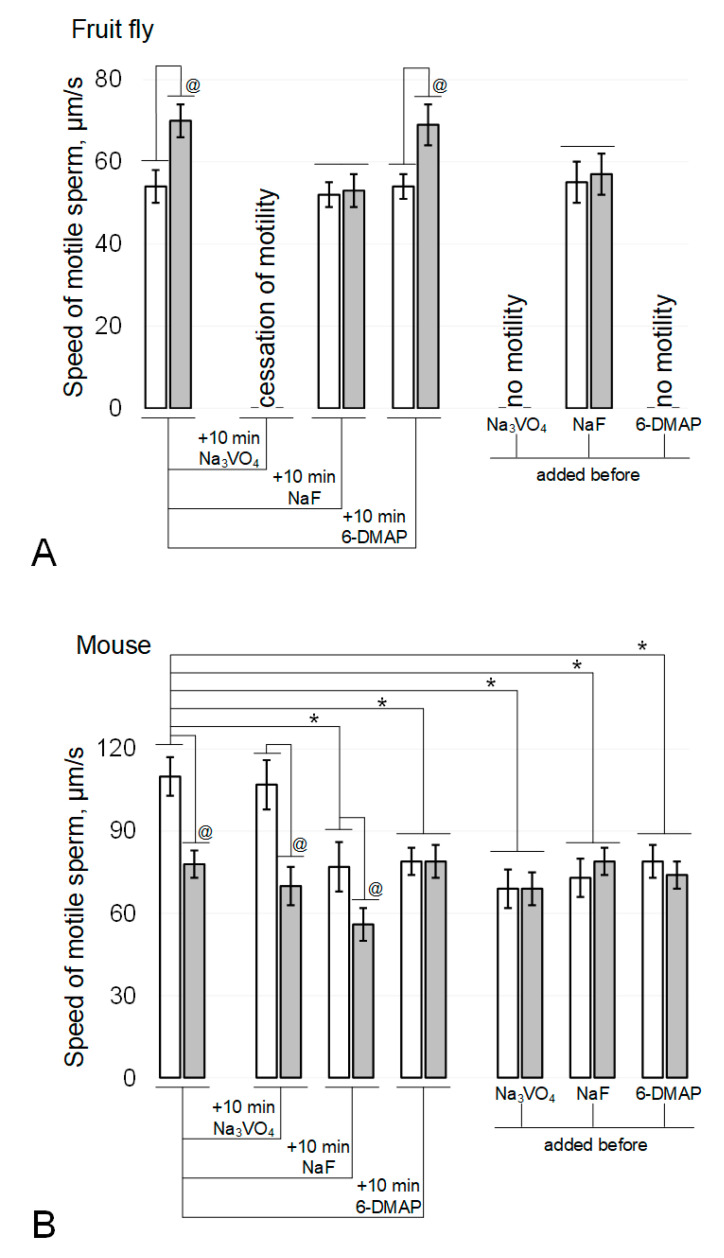
Sperm motility after 6-h modelling microgravity. (**A**) fruit fly Drosophila melanogaster. (**B**) mouse. White columns—control, grey columns—after 6 h exposure under modelling microgravity conditions. Na_3_VO_4_—200 µM sodium orthovanadate (Tyr phosphatase inhibitor). NaF—5 mM sodium fluoride (Ser/Thr phosphatase inhibitor). 6-DMAP—0.5 mM 6-(dimethylamino)purine (protein kinase inhibitor). @ *p* < 0.05 in comparison with according control group, * *p* < 0.05 in comparison with control without any inhibitors.

In the fruit fly Drosophila melanogaster, the rate of sperm movement after exposure to simulated microgravity (Figure 1A) increased by 30% (*p* < 0.05). The addition of 200 µM sodium orthovanadate (Na_3_VO_4_), an inhibitor of Tyr phosphatases, and incubation for 10 min after the end of the exposure under simulated microgravity conditions led to a complete cessation of motility in both the control and experimental groups. The addition of 5 mM sodium fluoride (NaF), an inhibitor of Ser/Thr phosphatases, and incubation for 10 min neutralized the effect of simulated microgravity without affecting movement speed in the control group. The addition of 0.5 mM 6-(dimethylamino)purine (6-DMAP), a broad-spectrum kinase inhibitor, and incubation for 10 min did not lead to changes in mobility relative to the initial parameters: in the control group, the speed did not change, and in the simulated microgravity group, it remained above the corresponding control by 28% (*p* < 0.05).

The addition of phosphatase inhibitors before the start of fly sperm exposure (Figure 1A) led to the same results as incubation after its termination: against the background of Na_3_VO_4_, there was no motility, and against the background of NaF, the effect of an increase in the speed of movement under simulated microgravity was not observed. However, exposure to a kinase inhibitor for 6 h resulted in complete cessation of sperm movement in both the control and experimental groups.

In contrast, exposure of mouse spermatozoa to simulated microgravity for 6 h (Figure 1B) resulted in a 29% decrease in their movement velocity (*p* < 0.05). The addition of sodium orthovanadate and incubation for 10 min did not change movement speed in either the control or experimental groups. The addition of sodium fluoride and incubation for 10 min led to the same decrease in movement speed in the control and experimental groups: in both cases, by 28% (*p* < 0.05). The addition of 6-DMAP and incubation for 10 min reduced the speed of movement in the control group by 27% (*p* < 0.05) but did not affect the speed of movement of sperm in the experimental group.

The addition of phosphatase and kinase inhibitors prior to exposure of mouse sperm (Figure 1B) resulted in a decrease in movement speed in the control groups (37% (*p* < 0.05) with the addition of 200 μM sodium orthovanadate, 34% (*p* < 0.05) with the addition of 5 mM sodium fluoride, and 28% (*p* < 0.05) when 0.5 mM 6-DMAP was added. However, in these cases, after exposure for 6 h under simulated microgravity, the speed of sperm movement was the same as in the corresponding control groups.

### 3.3. Protein Content

**Figure 2:**
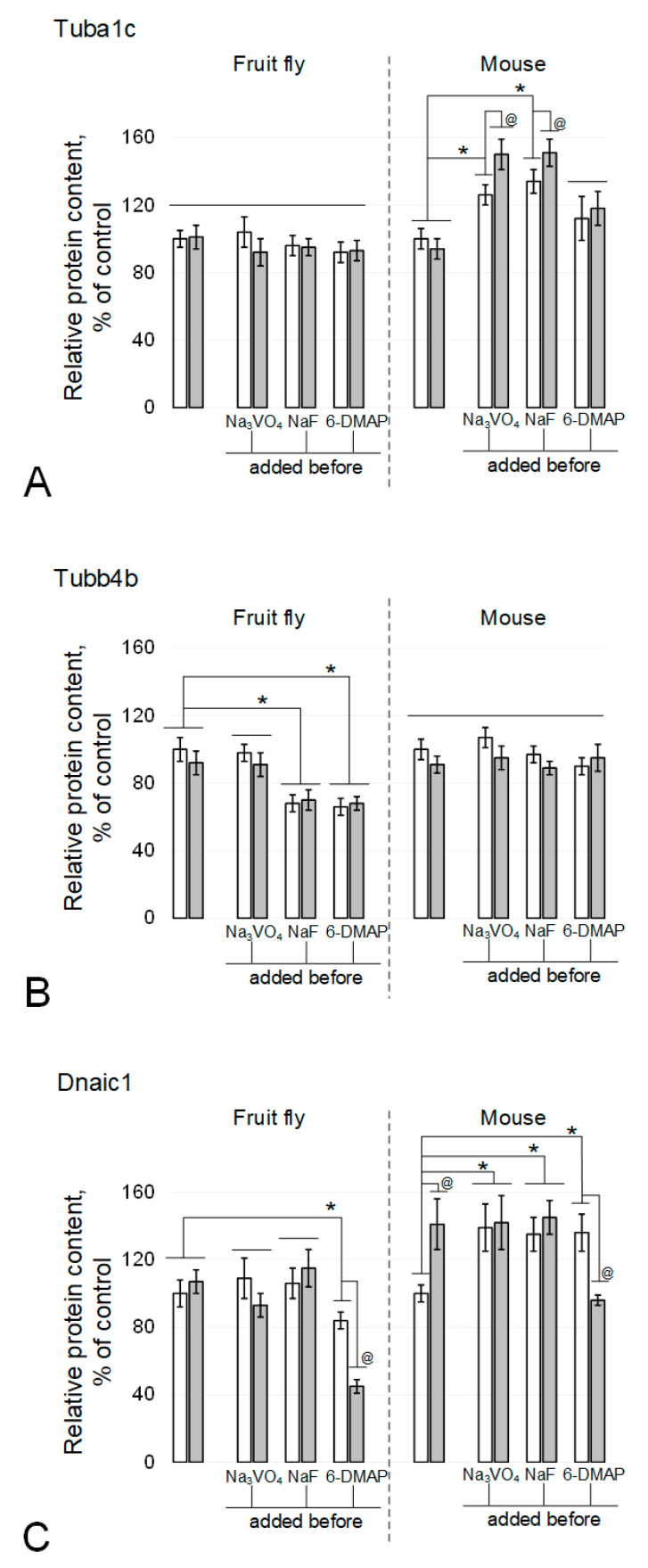
Tubulin isoforms and dynein relative content in the fly and mouse sperm. (**A**) alpha-tubulin Tuba1c. (**B**) beta-tubulin Tubb4b. (**C**) dynein Dnaic1. White columns—control, grey columns—after 6 h exposure under modelling microgravity conditions. @ *p* < 0.05 in comparison with according control group, * *p* < 0.05 in comparison with control without any inhibitors.

**Figure 3:**
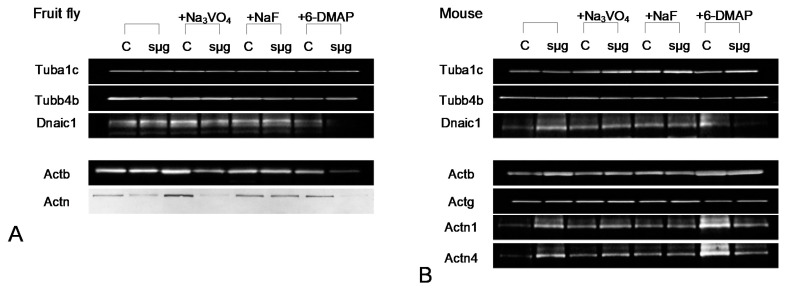
Typical western blot pictures. (**A**) *Drosophila melanogaster* proteins. (**B**) mouse sperm proteins.

The alpha-tubulin content in spermatozoa of the fruit fly Drosophila melanogaster did not change after 6 h of exposure under simulated microgravity (Figure 2A and Figure 3A), including in the presence of phosphatase and kinase inhibitors. In mice, exposure to the kinase inhibitor 6-DMAP also did not lead to changes in the relative content of alpha-tubulin (Figure 2A and Figure 3B). In the presence of phosphatase inhibitors, sodium orthovanadate and sodium fluoride, the relative content in the control increased by 26% (*p* < 0.05) and 34% (*p* < 0.05), respectively. Moreover, under simulated microgravity in the presence of sodium orthovanadate and sodium fluoride, the relative content of alpha-tubulin significantly increased relative to the corresponding controls and exceeded the initial level by 50% (*p* < 0.05) and 51% (*p* < 0.05), respectively.

In contrast, the content of beta-tubulin in mouse spermatozoa did not significantly change in the presence of inhibitors, although there was some downward trend after exposure under simulated microgravity, including sodium orthovanadate (Figure 2B and Figure 3B). In the fruit fly, no changes were noted under microgravity exposure with orthovanadate (Figure 2B and Figure 3A). Exposure to sodium fluoride and 6-DMAP led to a decrease in the relative content of beta-tubulin in the control groups by 32% (*p* < 0.05) and 34% (*p* < 0.05), respectively, which did not change after exposure to microgravity.

The relative dynein content in Drosophila melanogaster spermatozoa did not change under simulated microgravity conditions, including against the background of phosphatase inhibitors (Figure 2C and Figure 3A). The addition of the protein kinase inhibitor 6-DMAP to the medium led to a decrease in the relative content of dynein by 16% (*p* < 0.05) in the control group and to an even greater decrease, by 55% (*p* < 0.05), after a 6-h exposure to simulated microgravity. In mouse sperm, 6 h of simulated microgravity resulted in a 41% increase in the relative dynein content (*p* < 0.05) (Figure 2C and Figure 3B). The introduction of sodium orthovanadate and sodium fluoride led to an increase in the relative content of dynein by 39% (*p* < 0.05) and 35% (*p* < 0.05), respectively, but prevented its increase under microgravity conditions. Administration of the kinase inhibitor 6-DMAP, as well as phosphatase inhibitors, increased the relative content of dynein by 36% (*p* < 0.05); however, after exposure to simulated microgravity conditions, it decreased to the control level.

**Figure 4:**
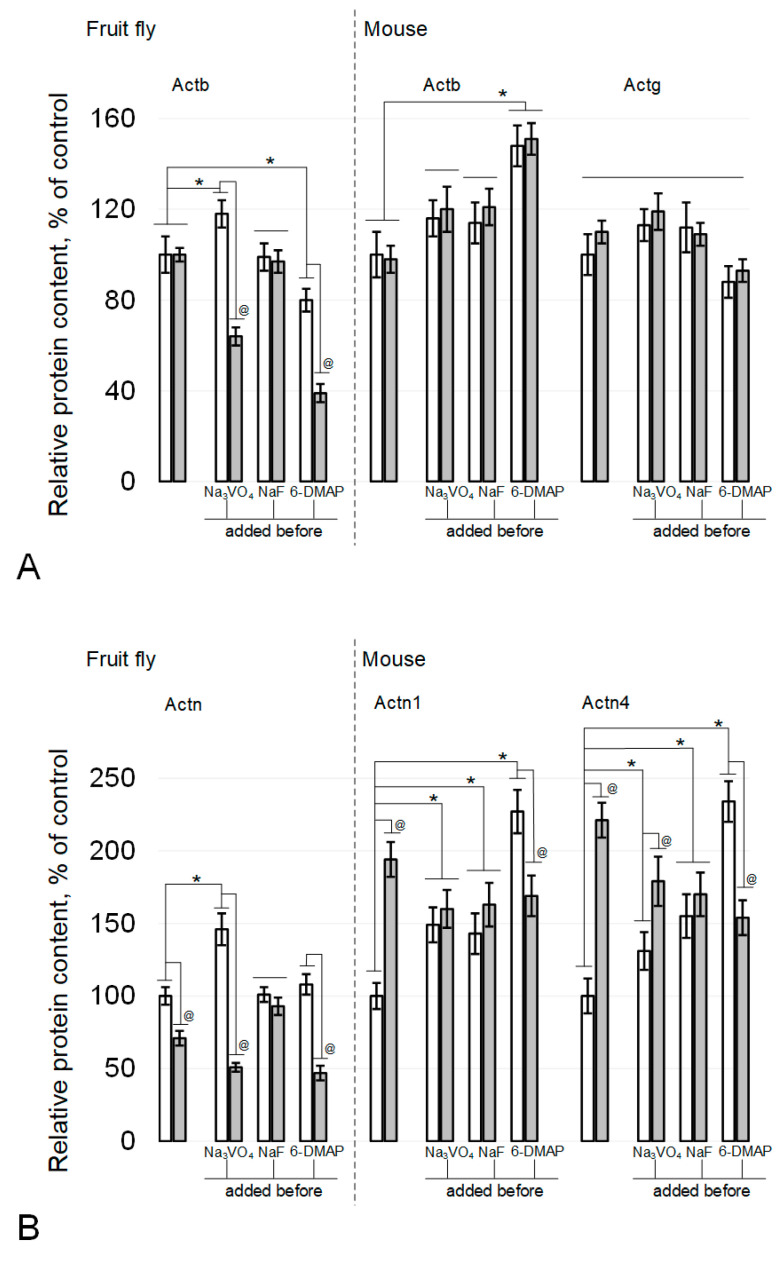
Actin and alpha-actinin isoforms relative content in the fly and mouse sperm. (**A**) actin isoforms, beta-actin Actb and gamma-actin Actg. (**B**) alpha-actinin isoforms, alpha-actinin Actn (*Drosophila melanogaster*), alpha-actinin1 Actn1 (mouse) and alpha-actinin4 Actn4 (mouse). White columns—control, grey columns—after 6 h exposure under modelling microgravity conditions. @ *p* < 0.05 in comparison with according control group, * *p* < 0.05 in comparison with control without any inhibitors.

The relative content of beta-actin in fly spermatozoa did not change under the influence of simulated microgravity or in the presence of sodium fluoride (Figure 3A and Figure 4A). The administration of sodium orthovanadate led to an increase in the content in the control by 18% (*p* < 0.05) and to a decrease by 36% (*p* < 0.05) after simulated microgravity. Against the background of 6-DMAP, the relative content of beta-actin decreased by 20% (*p* < 0.05) and even more significantly, by 61% (*p* < 0.05), after simulated microgravity. In sperm cells of mice, the relative content of beta-actin (Figure 3B and Figure 4A) remained at the control level after simulated microgravity, including during the addition of phosphatase inhibitors into the medium. The administration of a kinase inhibitor led to an increase in beta-actin content by 48% (*p* < 0.05), which did not change under simulated microgravity. In addition, the relative content of gamma-actin in mouse sperm remained at the same level in all study groups (Figure 3B and Figure 4A).

The content of actin-binding proteins changed differentially in fly and mouse spermatozoa under simulated microgravity conditions, including with the administration of inhibitors. Fruit fly alpha-actinin Actn (mouse Actn1 homologue) decreased by 29% (*p* < 0.05) after 6 h of exposure to simulated microgravity (Figure 3A and Figure 4B). The administration of sodium fluoride (inhibitor of Ser/Thr phosphatases) neutralized this effect without changing the relative content of Actn in the corresponding control. The administration of sodium orthovanadate (an inhibitor of Tyr phosphatases) increased the relative content of Actn in the control by 46% (*p* < 0.05), but under simulated microgravity, it decreased by 49% (*p* < 0.05) relative to the initial level. On the background of administration of 6-DMAP, the initial level of alpha-actinin did not change, but after exposure to microgravity conditions, it decreased by 53% (*p* < 0.05).

The relative alpha-actinin1 content in mouse sperm (fly Actn homologue) increased 94% (*p* < 0.05) after 6 h of simulated microgravity (Figure 3B and Figure 4B). The administration of sodium orthovanadate and sodium fluoride increased the relative content of Actn1 in the control by 49% (*p* < 0.05) and 43% (*p* < 0.05), respectively, but it did not change upon exposure to simulated microgravity. Against the background of 6-DMAP, the content of Actn1 in the control increased by 127% (*p* < 0.05), but after simulated microgravity, it decreased, exceeding the initial level by 69% (*p* < 0.05).

The relative content of another isoform, alpha-actinin4, in mouse sperm also increased after exposure to simulated microgravity for 6 h by 121% (*p* < 0.05) relative to the control (Figure 3B and Figure 4B). The introduction of orthovanadate increased the initial Actn4 content by 31% (*p* < 0.05), and even more significantly, it increased after simulated microgravity, exceeding the control level by 79% (*p* < 0.05). Against the background of sodium fluoride, the relative content of Actn4 also increased by 55% (*p* < 0.05) but remained at the same level after simulated microgravity. The introduction of 6-DMAP, as well as Actn1, led to an increase in the relative content of Actn4 in the control by 134% (*p* < 0.05), but after 6 h of exposure under simulated microgravity conditions, it decreased and exceeded the control level by 54% (*p* < 0.05).

## 4. Discussion

Human exploration of deep space and its preservation as a species is impossible without solving the problem of maintaining the health of the reproductive system. One of the particular issues that requires research and solution is the motor activity of spermatozoa, which is necessary for fertilization in vivo. Interestingly, various data from previous studies indicate that the motor activity of spermatozoa in mammals decreases under conditions of real or simulated microgravity [9,11,12], while in lower animals (sea urchin, fruit fly), it decreases [6,7,8]. At the same time, the proteins of the axoneme—the structure of the tail of the sperm, which ensures its movement -are rather conserved. According to BLAST NCBI data (https://blast.ncbi.nlm.nih.gov/Blast.cgi, accessed on September–November 2020), the homology of Drosophila melanogaster alpha-tubulin with mouse alpha-tubulin is 96.67%, beta-tubulin is 96.62%, and dynein is 47.63%. Accordingly, it can be assumed that the regulation of the activity of these proteins may be different. For example, changes in the motor activity of sea urchin spermatozoa under microgravity are likely due to changes in phosphorylation as a result of modulation of phosphatase activity [7]. Therefore, we decided to carry out a database search for phosphorylation regulators identified in fly and mouse spermatozoa.

**Figure 5:**
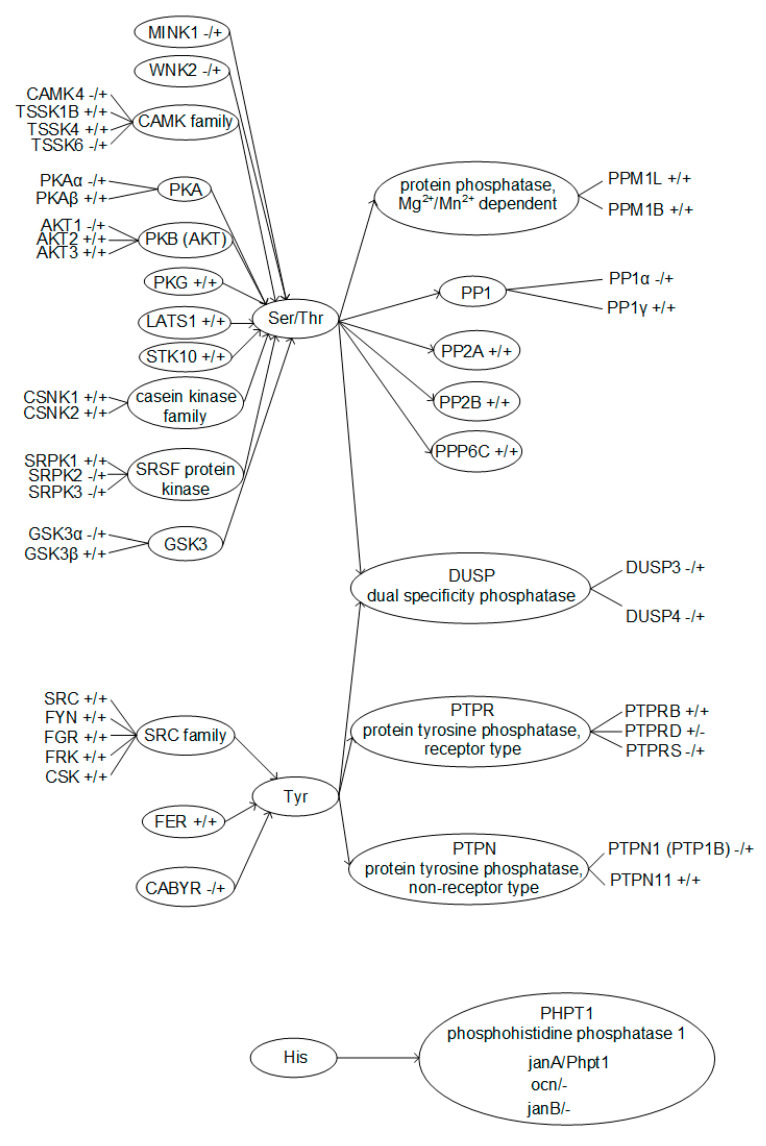
Phosphorylated amino acids residues with according kinases and phosphatases, identified in Drosophila melanogaster and mouse sperm. +/+ means existence of the protein in the Drosophila melanogaster/mouse genome, respectively.

A taxonomic search showed that different Ser/Thr kinases and phosphatases were identified in spermatozoa in both the fly and the mouse (Figure 5). Of the Ser/Thr phosphatases described in mouse spermatozoa, only PP1α has no homologue in Drosophila melanogaster. At the same time, for Ser/Thr protein kinases, the correspondences are significantly lower; in particular, PKAα, AKT1, and GSK3α have no homologue in flies. For the identified Tyr protein kinases and phosphatases, apart from CABYR and PTP1B, respectively, there are homologues in the mouse and fly. However, on the other hand, there are significantly fewer Tyr phosphorylation regulators described in spermatozoa. Moreover, phosphatases of dual specificity, that is, capable of dephosphorylating both Ser and Thr residues and Tyr residues, have been described only in mice. Phosphorylation of histidine is widespread in prokaryotes but rare in animals [31]. Possibly, the reduction in the evolutionary process of this mode of regulation is reflected in the fact that histidine phosphatase in the fly has 3 isoforms, while in the mouse there is only one, and it is not entirely clear whether it is expressed in spermatozoa.

Therefore, in view of the complex functional relationship of phosphorylation regulators, we carried out a direct experiment to identify the role of protein kinases and phosphatases in changing the motor activity of spermatozoa under microgravity conditions.

We observed an increase in the motor activity of Drosophila melanogaster spermatozoa after a 6-h exposure under simulated microgravity conditions. The addition of the protein kinase inhibitor 6-(dimethylamino)purine after exposure did not decrease the movement speed, which is consistent with the data obtained in sea urchin spermatozoa [7]. However, the addition of the Tyr phosphatase inhibitor led to a complete cessation of movement both in the control and after exposure under simulated microgravity, which may indicate that dephosphorylation of Tyr is absolutely necessary for motility under any conditions. Since the Ser/Thr phosphatase inhibitor neutralized the effect of microgravity and reduced the speed of movement of fly spermatozoa to the control level, while not affecting the speed of movement in the control in any way, we can cautiously assume that additional Ser/Thr dephosphorylating activity appears under microgravity conditions.

In contrast, in mice, after a 6-h exposure to simulated microgravity, the motor activity of spermatozoa decreased. Tyr phosphatase inhibitor did not affect the observed effect, so dephosphorylation of Tyr residues, in contrast to *Drosophila melanogaster*, is not a necessary condition for sperm motility. The administration of the Ser/Thr phosphatase inhibitor led to the fact that, the effect of reducing the speed persisted, but the initial level was lower. The addition of the protein kinase inhibitor 6-(dimethylamino)purine after exposure reduced the speed of movement in the control but did not affect the speed of movement after simulated microgravity. In other words, the protein kinase inhibitor had the same effect on the speed of movement of mouse sperm as simulated microgravity. This suggests that a decrease in protein kinase activity occurs under microgravity conditions.

On the other hand, it is well known that the use of phosphatase inhibitors induces the condensation of cortical actin in various cell types [32,33,34,35,36,37]. An increase in the stiffness of the cortical cytoskeleton makes it possible to prevent certain structural and functional changes in cells under simulated microgravity, in particular, changes in the content of cytoskeletal proteins [10,38]. Since we decided to conduct a second series of experiments with the administration of inhibitors before the start of exposure under modelling microgravity and estimate cytoskeletal proteins content.

In this case, an interesting fact was that for the fly, the effects on motility were similar to the introduction of inhibitors after simulated microgravity (with the exception of cessation of movement against the background of a kinase inhibitor). At the same time, for mouse spermatozoa, the speed of movement decreased in the control, but simulated microgravity had no effect.

Previously, we assumed that dissociation from the cortical cytoskeleton of actin-binding proteins, in particular, various isoforms of alpha-actinin, and a change in their content in the cytoplasm, may be the first stage of mechanoreception and lead to the triggering of signalling cascades [39,40]. Under microgravity conditions, there were no changes in the tubulin cytoskeleton and there were no changes in actin content. However, changes in the content of actin-binding proteins in the fly and mouse were different after 6 h of simulated microgravity. There is only one alpha-actinin isoform in the fly, as opposed to four in the mouse. The fly Actn homologue is mouse alpha-actinin1. After 6 h of simulated microgravity, the relative content of Actn in fly spermatozoa decreased, and Actn1 and Actn4 in mouse spermatozoa increased. Earlier, we observed a decrease in the total content of actin-binding proteins on different types of cells in the first stages upon receipt of a mechanical stimulus and an increase thereafter [41,42]. Therefore, it can be assumed that such an increase in both isoforms of alpha-actinin for mouse spermatozoa may indicate an already developed reaction and the formation of an adaptive protein pattern under new mechanical conditions. In other words, mechanotransduction in mouse spermatozoa proceeded further than in fly spermatozoa during 6 h of simulated microgravity. Moreover, the introduction of phosphatase inhibitors leads to an increase in the content of both actin itself and actin-binding proteins and, in addition to orthovanadate in flies, prevents changes in the content of alpha-actinin.

Summarizing the results obtained, we can conclude that after 6 h of simulated microgravity, the motor activity of *Drosophila melanogaster* spermatozoa increases. This effect can be neutralized by a Ser/Thr phosphatase inhibitor. For mouse spermatozoa, after 6 h of simulated microgravity, there is a decrease in motor activity. Protein kinase inhibitor reduces motor activity to the same level. Moreover, changes in the content of actin-binding proteins in fly and mouse spermatozoa were also multidirectional, which may indicate different mechanisms of gravireception and/or different rates of formation of the adaptive pattern of proteins.

This is a pilot study of the effect of different changes in sperm motility under simulated microgravity in evolutionarily distant animal species. The limitations of the study are associated with the wide spectrum of action of the inhibitors used. Subsequent studies of this issue should be accompanied by point inhibition of specific enzymes and determination of kinase/phosphatase activity. Analysis of the fine mechanisms of gravireception in the future may include not only the condensation of cortical actin, but also its disassembly.

## Data Availability

All data generated or analyzed during this study are included in this article.
